# Risk Assessment and Correlation Analysis of Potentially Toxic Element Pollution in Soil and Crops: A Case Study in a Typical Area

**DOI:** 10.3390/toxics13070554

**Published:** 2025-06-30

**Authors:** Jiufen Liu, Cang Gong, Yinji Ba, Shuliang Liu, Huiyun Wan, Xiaofeng Zhao, Ziqi Li, Xiaohuang Liu, Zhongfang Yang

**Affiliations:** 1 China University of Geosciences, Beijing 100083, China; 13863858360@163.com (J.L.); liziq@sina.com (Z.L.); 2 National Research Center for Geoanalysis, Key Laboratory of Eco-Geochemistry, Ministry of Natural Resources, Beijing 100037, China; 3 Key Laboratory of Natural Resource Coupling Process and Effects, Beijing 100055, China; zhaoxiaof@sina.com (X.Z.); liuxiaohuang@sina.com (X.L.); 4 Technology Innovation Center for Analysis and Detection of the Elemental Speciation and Emerging Contaminants, China Geological Survey, Kunming 650111, China; 5 Natural Resources Comprehensive Survey Command Center of China Geological Survey, Beijing 100055, China; 6 Research Center of Applied Geology of China Geological Survey, Chengdu 610036, China; 7 Yantai Center of Coastal Zone Geological Survey, China Geological Survey, Yantai 264000, China; bayinji@sina.com (Y.B.); liusl@sina.com (S.L.); wanhy@sina.com (H.W.)

**Keywords:** crops, potentially toxic elements, pollution risk, spatial differentiation, stepwise linear regression analysis

## Abstract

Soil contamination with potentially toxic elements (PTEs) not only poses potential ecological risks (RI) but also leads to human health risks (HI) through the uptake of potentially toxic elements by crops. However, most studies primarily focus on potentially toxic element contamination in either soil or crops, often neglecting the intrinsic connections between soil and crop contamination risks. In reality, some regions may exhibit severe soil PTE exceedances, yet the PTE levels in crops may not necessarily exceed regulatory limits, resulting in human health risks that are not uniformly high. This study investigated a typical area with severe soil PTE pollution caused by wastewater from electroplating, smelting, and ore beneficiation industries, and conducted risk assessments on soil and crops. The research aims to elucidate the differences in soil and crop PTE contamination risks and the correlations between PTE concentrations in soil and crops. Results showed that Cd was the most severe PTE contaminant in the soil in the study area, with an average concentration of 1.11 mg/kg and a maximum concentration of 7.30 mg/kg. However, the average concentrations of eight PTEs in crops were all below the standard limits for cereal crops specified in the Food Safety National Standard for Pollutant Limits in Foods (GB 2726-2022). Cd was identified as the most severe PTE contaminant in the soil, resulting in the highest RI (836) in the MY sub-region of the study area. However, Cr in crops contributed the most to health risk (63.5%), leading to the highest HI (7.1) in sub-region MY. Despite Cd being the most severely polluting PTE in soil, its contribution to human health risk through crops was relatively low, ranging from 2.82% to 9.90%. This discrepancy in pollution risks indicates that a PTE causing severe soil contamination may not necessarily result in significant human health risks via crop uptake. Correlation and regression analyses revealed that soil PTEs had the greatest impact on Cd levels in crops. Soil Ni, Cd, Cu, As, and Zn exhibited different synergistic or antagonistic effects on crop PTE uptake. Notably, soil Cd content showed a highly significant positive regression relationship with Cd, Cr, and Ni concentrations in crops. Overall, the influence of soil PTEs on crop PTEs varied significantly, and the spatial differentiation characteristics of PTEs in soil and crops differed. For PTEs with high spatial differentiation, localized and precise management measures should be implemented. Conversely, for PTEs with low spatial differentiation, unified risk management and control measures can be adopted.

## 1. Introduction

PTE contamination in soil has long posed a significant threat to ecosystems and human health on a global scale [1,2,3]. The accumulation of PTEs in soil originates from both natural and anthropogenic sources [1]. Human activities, including mining, metallurgical processes, chemical production, industrial emissions, and the use of treated wastewater for irrigation, play a major role in causing PTE pollution [4,5,6,7,8]. Over the past period, at least 30,000 tons of Cr and 800,000 tons of Pb have been released into the environment globally, with the majority infiltrating the soil and causing significant PTE contamination [1,9]. The national soil pollution survey revealed that 16.1% of soil samples failed to meet the environmental standards set by the Ministry of Ecology and Environment. Among the heavy metals identified, lead, cadmium, mercury, chromium, arsenic, nickel, and copper exhibited exceedance rates of 1.5%, 7.0%, 1.6%, 1.1%, 2.7%, 4.8%, and 2.1%, respectively. Notably, cadmium was the primary contributor to the highest potential ecological risks associated with soil contamination [10,11]. Additionally, soil PTE contamination has led to widespread PTE contamination in crops globally [12,13,14,15,16,17,18,19,20,21,22]. In China, more than half of the rice samples collected near active mining areas exceeded the Cd allowance limit specified by the Chinese food safety standards [23]. In India, 50% of vegetables grown in wastewater-irrigated fields showed significant accumulation of heavy metals [24]. PTEs in crops pose significant risks to human health through dietary exposure, as they accumulate in vital organs such as the lungs, kidneys, liver, bones, and gastrointestinal tract, leading to chronic conditions including diabetes, emphysema, nephrolithiasis, osteoporosis, renal tubular damage, male reproductive disorders, pregnancy-related illnesses, and various forms of cancer [7,8,13,14,24,25,26,27,28], thereby causing a variety of health issues.

The PTE content in crops and soil is interconnected. PTEs from contaminated soils can translocate to different tissues of wheat and accumulate within the plant [29]. Different crops exhibit varying abilities to absorb specific PTEs from soil, resulting in distinct accumulation patterns [30]. For instance, in rice, the accumulation capacity follows the order As < Cu < Zn < Cd [31], while crops such as cabbage, tomatoes, and lettuce demonstrate significantly higher absorption and accumulation capacities for Zn compared to Cd [32]. However, not all PTE contamination in crops originates from soil [33]. For example, PTEs from atmospheric particulates can enter crops through stomata on crop leaves [34], and irrigation with contaminated water is another significant source of PTE accumulation in crops [6].

The accumulation of PTEs from soil to plants is primarily dependent on absorption mechanisms, soil physicochemical properties, and the chemical forms of PTEs in the soil [35]. While existing studies have considered both soil and crop PTEs, they have rarely explored the relationships between the two. Risk assessments based solely on total metal concentrations in soil may overestimate the risks, potentially leading to unnecessary and costly soil remediation efforts. In reality, PTEs exhibit complex interaction relationships, such as synergistic and antagonistic effects. The simultaneous presence of multiple PTEs in soil inevitably influences crop PTE uptake during the process [36]. For example, the presence of Ni in soil can suppress the uptake of Cd and Cu by corn roots, whereas Ni and Cu in soil can stimulate the accumulation of Pb and Cu in corn stems. Moreover, Pb and Cr in soil can facilitate the accumulation of Mn in corn kernels [37]. While previous studies have attempted to elucidate the relationships between PTEs in soil and crops, their focus has predominantly been on specific PTE combinations in particular crops or on bioavailability and mobility within soil–plant systems, often relying on limited pot or field experiments. There is a lack of regional-scale studies investigating the interrelationships among multiple typical PTEs in soil–crop systems.

A typical region, as a major grain, cotton, and high-quality wheat production base in China, as well as a national livestock production and processing base, has experienced soil contamination due to industrial wastewater irrigation. This has led to elevated concentrations of PTEs such as Cd, Ni, Zn, and Cu in the soils of polluted irrigation areas [38]. To better understand the PTE contamination characteristics and correlations in the soil–crop system of this region, a total of 145 soil samples and 145 wheat samples were systematically collected. This study aims to investigate the differences in PTE contamination risks between soil and crops and explore the relationships between PTE concentrations in soil and crops.

## 2. Materials and Methods

### 2.1. Study Area and Sample Collection

**Figure 1:**
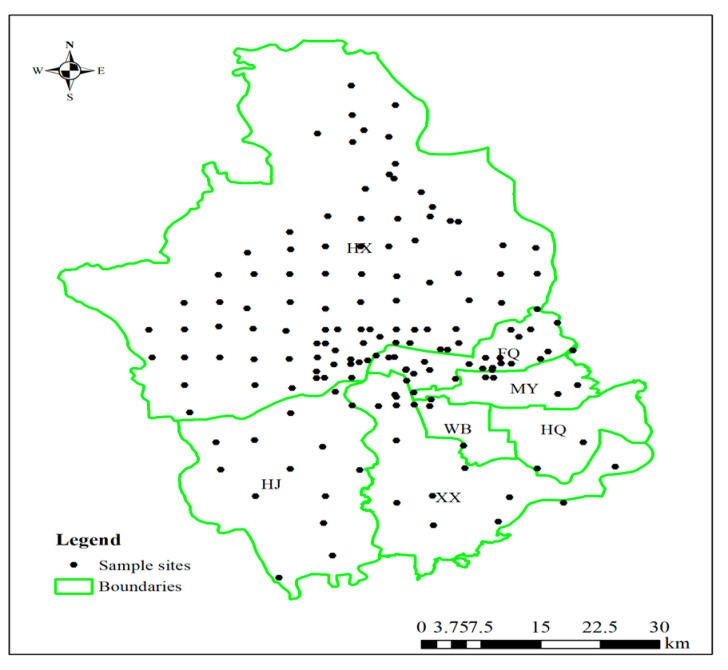
Geographical location of the study area and spatial distribution patterns of PTE samples.

The study area (113°04′35″–114°17′3″E, 35°50′57″–35°04′24″ N) (Figure 1) is situated within a warm temperate continental monsoon climate zone, characterized by a mean annual temperature of 14 °C and receiving 650 mm of annual precipitation. The topography slopes downward from northwest to southeast, with the northwest and northern regions characterized by low to medium mountains and hills. This region is a major grain and cotton production area in China and a national base for high-quality wheat production. However, in recent years, rapid urban expansion and development have led to the emergence of numerous storage battery manufacturing enterprises in the region. The production processes of these batteries generate wastes containing Cd and As, resulting in pollution of the atmosphere, soil, and surface water in the study area and its surrounding regions. Notably, the utilization of industrial effluents from electroplating, smelting, and ore beneficiation by local farmers for agricultural purposes has significantly increased PTE contamination levels, posing significant risks to both soil quality and crop safety.

Sampling was conducted across seven sub-regions (HX, HJ, XX, WB, HQ, MY, and FQ) within the study area, following the distribution of arable land. A grid method (5 m × 5 m) was employed to systematically collect soil and wheat samples, with a total of 145 sampling points established (Figure 1). Soil samples (0–20 cm depth) were obtained using a five-point sampling method, homogenized into composite samples, and treated to eliminate roots and other impurities. The samples were then transferred to sample bags using a wooden shovel, properly labeled, and securely stored until submission. At each sampling site, both soil and wheat samples were collected simultaneously, with detailed documentation of sample center coordinates, sampling date, and location. Upon return to the laboratory, soil samples were air-dried, residues and stones were removed, and the samples were ground and sieved through a 60-mesh sieve. The processed samples were then packaged in polyethylene bags and submitted to a third-party laboratory accredited with CMA/CNAS certification for analysis and detection of target pollutants. Soil samples underwent digestion employing a HNO_3_-HF-HClO_4_ acid system. The concentrations of Cu, Pb, Zn, Cr, Cd, and Ni were measured using inductively coupled plasma mass spectrometry (ICP-MS). As and Hg were digested using aqua regia under heated conditions and analyzed by atomic fluorescence spectrometry (AFS). Wheat samples were processed by removing impurities, grinding, and sieving through a 20-mesh screen. The PTE concentrations were determined in compliance with the “Food Safety National Standard” GB5009 series. Cd, Cr, Pb, and Ni concentrations were analyzed using graphite furnace atomic absorption spectrometry (GFAAS), while As and Hg concentrations were determined using AFS, and Cu and Zn concentrations were analyzed using flame atomic absorption spectrometry (FAAS). Each sample was analyzed in triplicate, with strict quality assurance and quality control measures implemented in accordance with the requirements of the analytical methods.

### 2.2. Research Methods

#### 2.2.1. Potential Ecological Risk Assessment

The Potential Ecological Risk Index (RI), introduced by Hakanson in 1980, has become a cornerstone in ecological risk assessment. The calculation formula is as follows:

(1) $$\mathrm{RI}=\sum_{i=1}^{n}E_{r}=\sum_{i=1}^{n}{(T}_{i}\times \frac{\;C_{i}}{\;B_{i}})$$

where RI is defined as the comprehensive potential ecological risk index for multiple PTEs at the sampling location, *i* represents a specific PTE, E*r* is the potential ecological risk index for a single PTE, and T*i* is the toxicity response coefficient for the corresponding PTE (As = 10; Zn = 1; Ni = 5; Cu = 5; Hg = 40; Pb = 5; Cr = 2; Cd = 30) [30]. C*i* is the measured content of the element. B*i* is the soil environmental quality standard for the PTE. In this study, the soil background values of the province where the study area is located were adopted as standards (As = 11.4 mg/kg; Zn = 60.1 mg/kg; Ni = 26.7 mg/kg; Cu = 19.7 mg/kg; Hg = 0.034 mg/kg; Pb = 19.6 mg/kg; Cr = 63.8 mg/kg; Cd = 0.074 mg/kg). E*r* can be classified into five levels based on its range: slight risk (E*_r_* < 40), mild risk (40 ≤ E*_r_* < 80), moderate risk (80 ≤ E*_r_* < 160), severe risk (160 ≤ E*_r_* < 320), and extremely severe risk (ERI ≥ 320). The RI can be classified into four levels based on its range: slight risk (RI < 150), mild risk (150 ≤ RI < 300), moderate risk (300 ≤ RI < 600), and severe risk (RI ≥ 600).

#### 2.2.2. Human Health Risk Assessment

The health risk index (HI) is used to evaluate human health risks associated with PTE intake through food consumption. When HI > 1, the intake of PTEs in crops poses a threat to human health, with higher coefficients indicating greater risks. The calculation formula is as follows:

(2) $${HI}_{i}=\frac{C_{i}\times IR\times EF\times ED}{BW\times AT\times {RfD}_{i}}$$

where HI*_i_* represents the non-cancer health risk index for PTE *i*. C*_i_* is the concentration of PTE *i* in crops. IR is the daily intake rate of cereal crops, with an adult intake rate of 0.293 kg/day [30]. EF is the exposure frequency, calculated as 365 days/year. ED is the exposure duration, set at 24 years [30]. BW is the body weight of the receptor, assumed to be 56.8 kg [30]. AT is the average exposure time, calculated as EF × ED. RfD*_i_* is the reference dose for PTE *i*. Based on USEPA standard parameters, the RfD*_i_* values for Cr, Cd, As, Hg, Pb, Cu, Zn, and Ni are 0.003, 0.001, 0.0003, 0.0003, 0.0035, 0.04, 0.3, and 0.02 mg/kg/day, respectively.

#### 2.2.3. Geographical Detector

Geographic detectors can be applied to spatial analysis across different domains, scales, and data types, including factor detectors, interaction detectors, risk zone detectors, and ecological detectors [39,40]. In this study, the factor detector was employed to analyze the spatial differentiation characteristics of potential ecological risks and human health risks associated with PTE pollution, with the degree of association measured by the *q* value. The calculation formula is as follows:

(3) $$q=1-\frac{\sum_{h=1}^{n}N_{h}\sigma_{h}^{2}}{N\sigma^{2}}=1-\frac{SSW}{SST}$$

where *h* = 1, …; N*_h_* and N represent the number of units in sub-region h and the entire study area, respectively. $\sigma_{h}^{2}$ and *σ*^2^ are the variances of sub-region h and the entire study area, respectively. SSW and SST represent the sum of variances within sub-regions and the total variance of the entire study area, respectively. The value of *q* ranges between 0 and 1, with a higher *q* indicating a greater degree of spatial differentiation. If the average risk index is high and *q* is large, this suggests a high spatial variability in the study area, necessitating localized and precise risk management measures tailored to specific conditions. Conversely, if the average risk index is high but *q* is small, this indicates low spatial variability, allowing for uniform risk management and control measures to be implemented across the study area.

#### 2.2.4. Rank-Size Theory

Based on the rank-size theory [41,42], this study combines the PTE risk assessment results at multiple sites within research sub-regions and the geographic detector analysis results. A model is employed to calculate the risk levels (R*_ij_*) of different sub-regions within the study area. The calculation formula is as follows:

(4) $$R_{ij}=\frac{\sum_{j=1}^{n}\frac{P_{j}}{\bar{P}}}{n}$$

In the formula, P*_j_* represents the risk value at sampling site *j* within sub-region *i*, P represents the mean risk value across the entire study area, and *n* represents the number of sampling sites within sub-region i*i*. The range of R*_ij_* can be classified into three levels: low risk level (R*_ij_* < 0.8), moderate risk level (0.8 ≤ R*_ij_* < 1), and high risk level (R*_ij_* ≥ 1) [30].

#### 2.2.5. Multivariate Statistical Analysis

##### Partial Correlation Analysis

Partial correlation analysis allows for the control of linear influences from other variables, thereby eliminating the effects of a third irrelevant element and focusing solely on the correlation between two target variables. By employing partial correlation analysis to examine the relationship between soil and crop concentrations of a specific PTE, the influence of other PTEs can be effectively controlled. If the partial correlation coefficient r is negative, it indicates an antagonistic relationship between the corresponding PTEs in soil and crops. Conversely, if r is positive, it suggests a synergistic effect between the PTEs in the soil and crop system.

##### Stepwise Linear Regression Analysis

In regression analysis, if there are two or more independent variables, it is referred to as multiple regression. When studying crops, the PTE content in crops is often influenced by multiple variables, making multiple linear regression analysis a suitable approach. The calculation formula is as follows:

*Y* = *β*_0_ + *β*_1_*X*_1_ + *β*_2_*X*_2_ + … + *β*_p_*X*_p_ + ε (5)

where Y represents the PTE content in crops. *β*_0_ is the intercept term, *β*_1_, *β*_2_, …, *β*_p_ are the regression coefficients, and ε represents the random error term. *X*_1_, *X*_2_, …, *X*_p_ are the independent variables.

In linear regression analysis, when a new variable is introduced, an F-test is used to assess the significance of all variables in the regression equation. The F-value estimates the overall significance of the regression model, i.e., the linear relationship between the dependent variable and all independent variables. If the F value is not significant and the probability (*p* value) exceeds the predetermined significance level, the variable is excluded, ensuring that only variables with significant effects are retained. This process of variable inclusion and exclusion is iterated until all independent variables in the regression equation significantly influence the dependent variable. The coefficient r represents the goodness of fit, indicating how well the estimated model aligns with the observed data. A value of r closer to 1 signifies a better fit of the model.

### 2.3. Data Processing

Descriptive statistical analysis of the data was conducted using SPSS 26.0. Sampling maps and spatial distribution maps were created using ArcGIS 10.8. Graphs were completed using Origin 2019. Geographic detector analysis was performed using GeoDetector software (http://www.geodetector.cn/index.html, accessed on 27 May 2025).

## 3. Results and Discussion

### 3.1. Statistical Analysis of PTEs in Soil and Crops

#### 3.1.1. Statistical Analysis of PTEs in Soil

The statistical results of PTE contents in the study area soils are presented in Table 1. The average concentrations of As, Cd, Cr, Cu, Hg, Ni, Pb, and Zn in the soils were 10.6, 1.11, 65.1, 30.1, 0.066, 29.9, 28.2, and 88.5 mg/kg, respectively. The average concentrations of Cd, Cr, Cu, Hg, Ni, Pb, and Zn in the study area soils were significantly higher than the soil background values (Table 1), particularly with the average Cd concentration being 15 times higher than the background value of 0.074 mg/kg, indicating a pronounced enrichment of PTEs (especially Cd) in the study area soils. Many studies have shown that the coefficient of variation is proportional to the degree of interference from external factors such as human activities [43]. The coefficients of variation for Cd and Hg in the study area soils were 107% and 110%, respectively, suggesting significant external interference. Considering the phenomenon of agricultural irrigation using industrial wastewater in the study area, the increase in Cd and Hg concentrations in the soils may be attributed to the irrigation with contaminated wastewater. Compared to the soil pollution risk screening values (GB 15618-2018), the concentrations of PTEs other than Cd were below the screening values. However, the average Cd concentration was 3.7 times higher than the risk screening value of 0.30 mg/kg, with Cd concentrations exceeding the screening value at 97.9% of the sampling sites. Notably, 19.3% of the sampling sites had Cd concentrations exceeding the soil pollution risk control value of 1.5 mg/kg. These findings indicate a significant Cd pollution risk in the study area soils.

#### 3.1.2. Statistical Analysis of PTEs in Crops

The statistical results of the PTE content in crops from the study area are presented in Table 2. The average concentrations of As, Cd, Cr, Cu, Hg, Ni, Pb, and Zn in crops were 0.056, 0.092, 1.30, 4.00, 0.0017, 0.58, 0.11, and 22.5 mg/kg, respectively. Compared to the limit values for cereals in the “Food Safety National Standard-Maximum Levels of Contaminants in Foods” (GB 2726-2022), the average concentrations of all PTEs in crops were below the standard limits. However, Cr, Hg, Cd, Ni, and Pb exceeded the standard limits in 73, 40, 29, 13, and 3 crop samples, respectively. Although the Cd sampling points exceedance rate in soils of the study area was 97.9%, the overall Cd sampling points exceedance rate in crops was only 20.0%, indicating a significant discrepancy between soil and crop Cd pollution evaluation results.

### 3.2. Risk Assessment of Soil PTE Pollution

#### 3.2.1. Potential Ecological Risks of Soil PTEs

**Figure 2:**
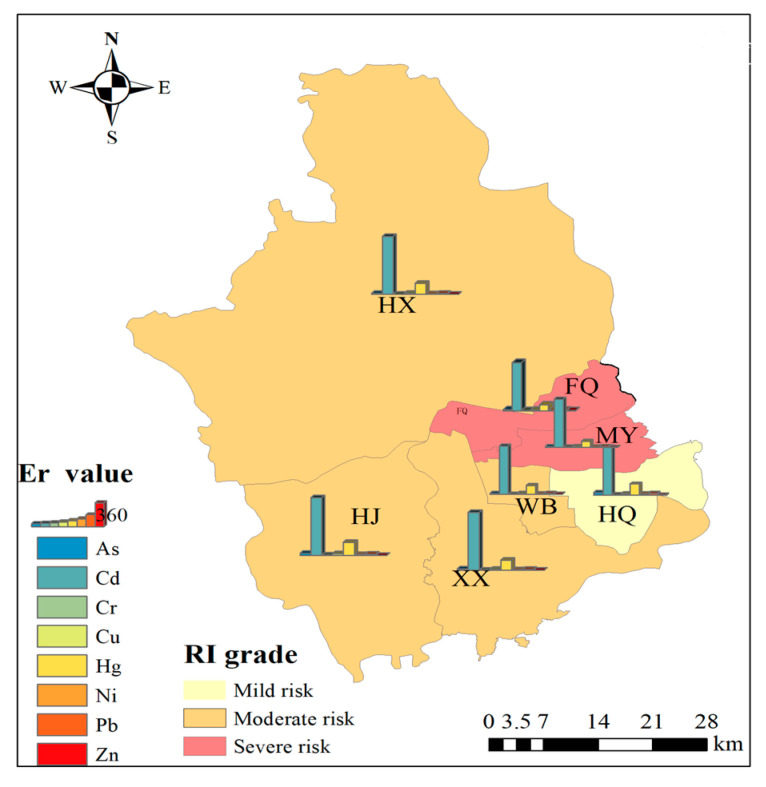
Potential ecological risk of soil PTEs.

The potential ecological risk of PTEs in the study area soils is shown in Figure 2, with the overall risk level being relatively high. The risk index (E*r*) for Cd was the highest, ranging from 189 to 711, indicating severe and extremely severe ecological risks. Hg followed, with an E*r* range of 43.6 to 85.4, corresponding to mild and moderate ecological risks. The maximum E*r* values for both Cd and Hg were observed in the MY sub-region. For the remaining PTEs (Cr, Pb, As, Cu, Zn, and Ni), the E*r* values were all below 11, indicating a low ecological risk level. Overall, Cd and Hg were the primary contributors to the comprehensive ecological risk in the study area, necessitating the implementation of appropriate management measures to address this issue.

#### 3.2.2. Spatial Differentiation Characteristics of Potential Ecological Risks

The potential ecological risks in different sub-regions of the study area show significant differences (Figure 2). Sub-regions FQ and MY are at a severe risk level, while HX, HJ, XX, and WB are at a moderate risk level, and HQ is at a mild risk level. The order of potential ecological risk levels among the sub-regions is MY > FQ > HX > XX > WB > HJ > HQ. The spatial differentiation characteristics of potential ecological risks, as analyzed by the geographic detector (*q*), vary significantly among the sub-regions, indicating pronounced differences in the potential ecological risks of soil PTEs across the study area (Table 3). The *q* values for potential ecological risks range from 0.018 to 0.13. Among the PTEs, the *q* values from highest to lowest are Cr > Ni > As > Pb > Cu > Zn > Cd > Hg. Obviously, Cr, Pb, As, Cu, Zn, and Ni exhibit relatively low potential ecological risks, have higher *q* values, reflecting distinct spatial differentiation characteristics. Therefore, localized management measures tailored to specific sub-regions are sufficient for these PTEs. Conversely, Cd and Hg have the smallest *q* values; despite their high average potential ecological risk index values, their spatial differentiation characteristics are not pronounced, suggesting that all sub-regions in the study area have experienced varying degrees of irrigation with industrial wastewater. As a result, uniform risk management measures across the study area are necessary.

The geographic detector serves as a robust analytical framework for examining the spatial heterogeneity of variables and their variability across spatial scales. It has found extensive application across a range of disciplines, including groundwater studies [44], land use [45], and ecological fragility [46]. In this study, the geographic detector was used to describe the spatial features of PTE pollution risks. This not only enriches the practical applications of the geographic detector but also provides supportive spatial information for subsequent risk level analyses.

#### 3.2.3. Ecological Risk Level of Soil PTEs Based on Rank-Size

**Figure 3:**
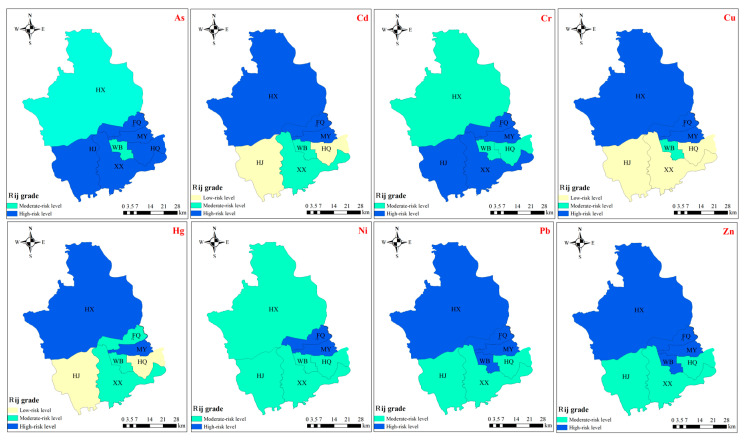
Potential ecological risk level of soil PTEs in different sub regions based on rank-size rule method.

Rank-size distribution can effectively reflect the spatial agglomeration characteristics of variables [47]. Based on the rank-size theory, the risk levels of soil PTEs in different sub-regions of the study area are shown in Figure 3. Except for sub-region HQ, As and Cr exhibited similar spatial agglomeration characteristics, with moderate risk levels in the north and high risk levels predominantly in the south. The spatial agglomeration characteristics and risk levels of Pb and Zn were identical, and their distribution was opposite to that of As and Cr. Sub-regions FQ and MY in the eastern part of the study area were high-risk zones for Ni. In terms of risk levels for Cd, Cu, and Hg, the north exhibited significantly higher levels than the south. Sub-regions HJ and HQ were low-risk zones for Cd and Hg, while HJ, XX, and HQ were low-risk zones for Cu.

### 3.3. Risk Assessment of PTE Pollution in Crops

#### 3.3.1. Human Health Risks of PTEs in Crops

**Figure 4:**
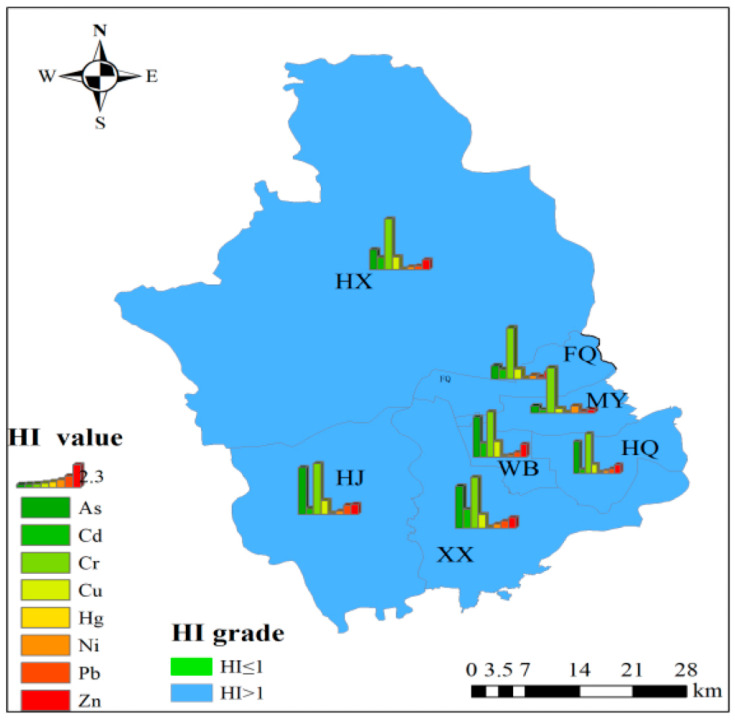
Human health risk of crop PTEs.

Due to the significant contributions of Cr and As, the HI for PTEs in crops from the study area was consistently above 1 (Figure 4), indicating that long-term consumption of these crops in large quantities may pose significant health risks to humans due to the PTEs they carry. Cr contributed the most to human health risks, with contributions ranging from 22.4% to 63.5%. As was the second-largest contributor, with contributions ranging from 9.55% to 22.6%. In contrast, the health risk contributions from PTEs such as Pb, Cd, Hg, Cu, Zn, and Ni were all below 10%. Although the soil Cd content in the study area ranged from 0.20 to 7.30 mg/kg (Table 1), the contribution of Cd to human health risks in crops was relatively low, ranging from 2.82% to 9.90%. Hg had the smallest contribution to health risks, with values not exceeding 0.8%. Overall, the health risk levels posed by PTEs in crops to human health in the study area were ranked as follows: Cr > As > Cu > Cd > Zn > Ni > Pb > Hg.

Human exposure to PTEs through the consumption of crops represents an indirect pathway of harm to human health. In addition to this, PTEs in soils can directly threaten human health through inhalation, dermal absorption, and soil ingestion [48]. In this study, direct human health risks were not considered, primarily because the amount of PTEs entering the human body directly via soil is extremely low, accounting for less than 1% of the intake through crops [49]. The consumption of crops is the primary route by which humans are exposed to PTEs.

#### 3.3.2. Spatial Differentiation Characteristics of Human Health Risks

Significant human health risks were observed in all seven sub-regions of the study area, with the combined contributions of Cr and As to human health risks ranging from 40.6% to 73.1% (Figure 4). The human health risk levels among sub-regions ranked as follows: MY > XX > FQ > HJ > WB > HQ > HX. The spatial differentiation characteristics (*q*) of human health risks, as analyzed by the geographic detector, varied significantly among sub-regions (Table 4). This indicates pronounced differences in the human health risks posed by PTEs in crops across the study area. The *q* values for human health risks ranged from 0.029 to 0.38. Among the PTEs, the *q* values ranked from highest to lowest were As > Hg > Pb > Ni > Cu > Zn > Cr > Cd. Overall, Cr exhibited particularly high average human health risk index values, but its *q* value among the eight PTEs was relatively small, with less pronounced spatial differentiation. Therefore, implementing uniform risk management and control measures across the study area is feasible. In contrast, As had the relatively largest *q* value, given its relatively high average human health risk index values, its distinct spatial differentiation characteristics necessitate localized and precise management measures to address the associated risks.

#### 3.3.3. Human Health Risk Level Based on Rank-Size

The rank-size rule was originally developed to explore the relationship between city hierarchy and scale [41]. In this study, the method was extended to the ecological and environmental fields. By combining spatial differentiation results, the rank-size rule was used to evaluate the risk sequences of sub-regions based on multiple soil and crop PTE data points, providing an integrated assessment of the ecological risks of soil PTEs and the health risks of crop PTEs across different sub-regions in the study area.

**Figure 5:**
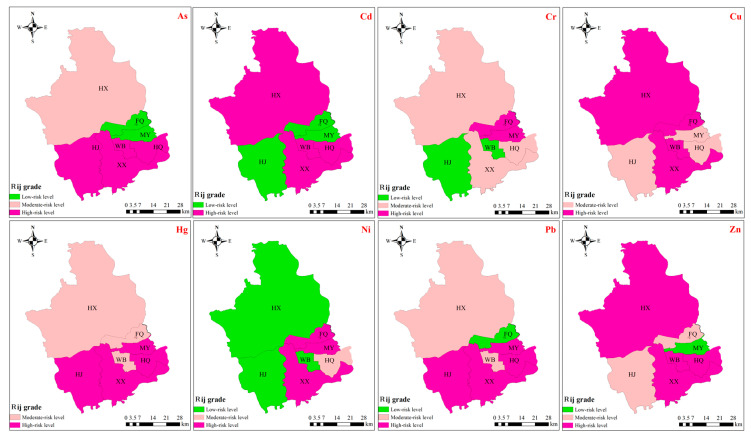
Human health risk level of crop PTEs in different sub regions based on rank-size rule method.

As shown in Figure 5, the risk levels of PTEs in crops across different sub-regions, based on the rank-size rule, exhibit distinct spatial patterns. As, Hg, and Pb demonstrated similar spatial aggregation characteristics and risk levels, with higher human health risk levels in the southern sub-regions compared to the northern ones. For Cd, Cu, and Zn, high-risk areas were concentrated in the northern and southern sub-regions, specifically in XX and WB. Cr exhibited high risk levels in sub-regions FQ and MY, with low risk levels in HJ and WB. Ni showed high risk levels in FQ, MY, and XX, adjacent to several low-risk sub-regions. These findings highlight the spatial variability in PTE risks across the study area.

### 3.4. Relationship Between PTE Content in Soil and Crops

Among the analyzed elements, Cd and Hg exhibit the highest potential ecological risks in soil, although their impact on human health risks is comparatively minimal. In contrast, Cr and As in crops present the greatest threat to human health, despite their ecological risks in soil being relatively low (as illustrated in Figure 2 and Figure 4). Furthermore, the risk levels associated with the eight PTEs demonstrate notable spatial variations. Significantly, the ecological and health risk levels associated with Cr and Hg show marked variations across different sub-regions. Importantly, there is a discrepancy between the soil ecological risks posed by PTEs and the human health risks linked to crop contamination, which can be attributed to the influence of various external factors, including fertilization, irrigation, tillage, and precipitation. The PTE content in crops is not only determined by the concentration of PTEs in the soil and the crops’ absorption capacity [12,50,51], but it is also closely related to soil properties such as pH, organic matter, cation exchange capacity, and PTE speciation [52,53,54,55,56,57]. While foliar absorption is a minor pathway for PTE uptake, root uptake is the primary mechanism by which crops absorb PTEs. Therefore, the PTE content in crops is ultimately determined by the concentration of PTEs in the soil.

#### 3.4.1. Correlation Between PTE Content in Soil and Crops

Table 5 summarizes the correlation analysis between PTE concentrations in soil and crops, revealing significant associations between these variables. The strongest influence on crop PTE concentrations was observed for Cd, followed by Ni. Specifically, Cd in crops exhibited significant positive correlations with Cd (R = 0.777), Ni (R = 0.344), and Cr (R = 0.242) in soil, indicating that these soil elements promote Cd uptake by crops. Similarly, Ni in crops showed significant relationships with Cd and As in soil, while Cr in crops was significantly correlated with Cd in soil. Additionally, Zn in crops was significantly correlated with Zn in soil. However, Hg, Cu, Zn, As, and Pb in crops did not demonstrate significant correlations with soil PTE concentrations.

#### 3.4.2. Regression Analysis of PTE Content in Soil and Crops

At the significant level, the contents of PTEs Hg, Cr, Ni, Zn, As, and Cd in crops (excluding Cu) exhibited varying degrees of linear regression relationships with PTEs in soil (Table 6). Overall, the synergistic or antagonistic effects of soil PTEs on crop PTEs were primarily concentrated on Ni, Cd, Cu, As, and Zn. Specifically, Ni in soil exhibited antagonistic effects on Hg, As, and Cd in crops. Cd in soil showed synergistic effects on Cr, Ni, and Cd in crops. Cu in soil exhibited antagonistic effects on the uptake of Zn and Pb by crops, while As in soil displayed significant synergistic effects on the uptake of Hg and As by crops. Zn in soil showed a synergistic effect on the uptake of Zn by crops. Notably, the content of Cd in crops exhibited an extremely significant positive regression relationship with Cd in soil, indicating a synergistic effect of soil Cd on the uptake of Cd by crops. A similar situation was observed for Zn and As.

The processes of PTE uptake, accumulation, translocation, and transformation in crops are not only influenced by the background concentration of PTEs in the environment [58] but also depend on various factors, including crop species, soil physicochemical properties, topographical characteristics, geochemical features, and the chemical speciation of PTEs [59,60,61]. Additionally, the differing external morphology and internal structure of crops result in distinct mechanisms for PTE absorption [62].

## 4. Conclusions

This study investigates the relationship between soil PTEs and crops by integrating the potential ecological risks of soil PTEs and the human health risks posed by PTEs in crops. For soil PTEs, the elevated ecological risk values of Cd and Hg result in the overall study area being classified under a high comprehensive ecological risk level. In contrast, PTEs such as Cr, Pb, As, Cu, Zn, and Ni exhibit relatively low potential ecological risks, with significant spatial differentiation, suggesting that region-specific management measures are sufficient. However, Cd and Hg, which demonstrate pronounced high potential ecological risks, exhibit minimal spatial differentiation (low *q* values), warranting uniform risk control measures across the entire study area. Spatially, the northern part of the study area is identified as a high-risk zone, while the overall study area is categorized under a medium–high risk level, consistent with the spatial distribution of high ecological risks.

For PTEs in crops, Cr and As exhibit significant contributions, resulting in a health risk index (HI) consistently above 1, indicating a high risk of human health hazards. Notably, despite Cd and Hg being the most polluted PTEs in soil, their contributions to human health risks are relatively low. The relatively small spatial differentiation of Cr in crops (*q* = 0.057) allows for uniform risk management measures across the study area, despite its high health risk. In contrast, As in crops exhibits a relatively large spatial differentiation (*q* = 0.38), necessitating localized and precise management strategies. In terms of health risk levels, the FQ and MY sub-regions are identified as high-risk zones for Cr, while the southern sub-regions are high-risk zones for As. The inconsistency in the content of the same PTE in soil and crops leads to distinct interaction relationships. The findings reveal that Cd in crops is most significantly influenced by soil PTEs, followed by Ni. In terms of crop absorption, the synergistic or antagonistic effects of soil PTEs on crop PTEs are primarily observed for Ni, Cd, Cu, As, and Zn. Notably, the contents of Cd, Cr, and Ni in crops exhibit extremely significant positive regression relationships with Cd in soil, suggesting that the elevated Cd content in soil promotes the uptake of Cd, Cr, and Ni by crops through a synergistic effect. Similar patterns are observed for Zn and As.

## Figures and Tables

**Table 1:** Descriptive statistical results of soil PTEs.

	As	Cd	Cr	Cu	Hg	Ni	Pb	Zn
Minimum (mg/kg)	5.50	0.26	43.4	15.3	0.0092	18.1	18.7	46.7
Maximum (mg/kg)	17.1	7.30	87.7	94.7	0.67	42.6	55.8	247
Mean (mg/kg)	10.6	1.11	65.1	30.1	0.066	29.9	28.2	88.5
Standard deviation (mg/kg)	2.58	1.19	8.27	11.6	0.073	5.16	5.09	29.9
Median (mg/kg)	10.5	0.70	65.1	28.1	0.047	29.8	28.0	81.4
Coefficient of variation (%)	24.4	107	12.7	38.6	110	17.2	18.1	33.8
Risk screening values for China soil contamination (GB 15618-2018, 6.5 < pH ≤ 7.5) (mg/kg)	30	0.30	200	100	2.40	100	120	250
Background values of soil (mg/kg)	11.4	0.074	63.8	19.7	0.034	26.7	19.6	60.1

**Table 2:** Descriptive statistical results of crop PTEs.

	Hg	Cr	Ni	Cu	Zn	As	Cd	Pb
Minimum (mg/kg)	0.00065	0.32	0.11	1.82	4.91	0.022	0.011	0.024
Maximum (mg/kg)	0.0038	11.7	14.3	6.82	39.5	0.19	1.04	0.55
Mean (mg/kg)	0.0017	1.30	0.58	4.00	22.5	0.056	0.092	0.11
Standard deviation mg/kg)	0.00085	1.38	1.25	0.73	5.50	0.032	0.14	0.090
Median (mg/kg)	0.0014	1.00	0.33	3.90	21.6	0.049	0.051	0.084
Coefficient of variation (%)	49.0	107	216	18.4	24.4	56.3	153	78.6
Limit of each potentially toxic element in cereals (mg/kg) (NY 861-2004, GB 2762-2022)	0.02	1.00	1.00	10.0	50.0	0.70	0.1	0.4

**Table 3:** Mean index values and spatial differentiation degrees of potential ecological risk of soil PTEs.

	As	Cd	Cr	Cu	Hg	Ni	Pb	Zn
FQ	10.6	538	2.16	8.11	64.7	6.27	7.23	1.63
HQ	9.91	189	2.03	6.08	43.6	5.37	7.13	1.31
HX	8.73	456	1.98	7.78	85.4	5.54	7.29	1.48
HJ	9.44	244	2.07	7.33	55.2	5.33	6.68	1.45
MY	10.4	711	2.31	10.0	85.4	6.25	8.34	1.65
WB	8.22	416	1.82	7.07	75.6	4.90	7.68	1.50
XX	9.95	423	2.12	5.86	73.2	5.23	6.36	1.18
*q*	0.11	0.039	0.13	0.072	0.018	0.12	0.093	0.054

**Table 4:** Mean index values and spatial differentiation degrees of potential ecological risk of crop PTEs.

	As	Cd	Cr	Cu	Hg	Ni	Pb	Zn
FQ	0.71	0.50	2.83	0.53	0.024	0.18	0.090	0.38
HQ	1.47	0.18	1.84	0.42	0.057	0.13	0.21	0.40
HX	0.81	0.49	2.08	0.53	0.026	0.11	0.15	0.40
HJ	1.61	0.20	1.75	0.48	0.045	0.11	0.32	0.34
MY	0.68	0.30	4.53	0.42	0.038	0.69	0.17	0.30
WB	1.41	0.48	1.60	0.54	0.028	0.062	0.15	0.45
XX	1.60	0.71	1.93	0.52	0.039	0.16	0.26	0.40
*q*	0.38	0.029	0.057	0.074	0.26	0.13	0.24	0.069

**Table 5:** Partial correlations between PTE contents in crops and soil.

	As_soil_	Cd_soil_	Cr_soil_	Cu_soil_	Hg_soil_	Ni_soil_	Pb_soil_	Zn_soil_
Hg_corp_	−0.058	−0.096	−0.146	−0.238	−0.062	−0.327	−0.167	−0.148
Cr_corp_	0.143	0.177 *	0.099	0.059	0.042	0.124	0.045	−0.011
Ni_corp_	0.168 *	0.217 **	0.115	0.032	0.02	0.117	0.001	−0.012
Cu_corp_	−0.119	−0.004	−0.087	0.097	0.064	−0.042	−0.028	0.079
Zn_corp_	−0.007	−0.031	−0.076	0.018	0.086	−0.068	−0.028	0.206 *
As_corp_	0.091	−0.163	−0.043	−0.121	0.036	−0.281	−0.171	−0.056
Cd_corp_	0.058	0.777 **	0.242 **	0.126	0.15	0.344 **	0.151	0.063
Pb_corp_	−0.033	−0.211	−0.101	−0.298	−0.057	−0.29	−0.139	−0.167

Note: ** represents a bilaterally significant correlation at 1% level, i.e., *p* < 0.01. * represents a bilaterally significant correlation at 5% level, i.e., *p* < 0.05.

**Table 6:** Stepwise linear regressions between PTE contents in crops and soil.

PTEs	Regression Equations	F Values	Adjusted R^2^	*p* Values
Hg	Hg_corp_ = −0.00009Ni_soil_ + 0.00011As_soil_ + 0.003	13.9	0.15	0.00
Cr	Cr_corp_ = 0.21Cd_soil_ + 1.07	4.63	0.025	0.00
Ni	Ni_corp_ = 0.23Cd_soil_ + 0.32	7.06	0.040	0.00
Zn	Zn_corp_ = 0.075Zn_soil_ − 0.13Cu_soil_ + 19.85	6.08	0.066	0.00
As	As_corp_ = −0.004Ni_soil_ + 0.007As_soil_ + 0.11	22.46	0.23	0.00
Cd	Cd_corp_ = 0.092Cd_soil_ − 0.001Zn_soil_ − 0.004Ni_soil_ − 0.043	81.16	0.63	0.00
Pb	Pb_corp_ = −0.002Cu_soil_ + 0.18	13.94	0.082	0.00

## Data Availability

All data generated or analyzed during this study are included in this published article.
